# Driver gene alterations profiling of Chinese non‐small cell lung cancer and the effects of co‐occurring alterations on immunotherapy

**DOI:** 10.1002/cam4.4178

**Published:** 2021-10-02

**Authors:** Shengjie Sun, Wenjuan Du, Qiong Sun, Xiao Zhao, Boyu Qin, Duozhi Shi, Chong Wan, Zhiyong Wu

**Affiliations:** ^1^ Medical Oncology Department The Fifth Medical Center of Chinese PLA General Hospital Beijing China; ^2^ Training Department of Medical Service Department General Hospital of People’s Liberation Army Beijing China; ^3^ Department of Medicine Lifehealthcare Clinical Laboratories Hangzhou China

**Keywords:** co‐occurring, driver genes, *EGFR*, immunotherapy, non‐small cell lung cancer, somatic alterations

## Abstract

**Background:**

Molecular testing for alterations in oncogenic driver genes and targeted therapies have become standard procedures for non‐small cell lung cancer (NSCLC) patients. However, little evidence has shed light on the pattern of co‐existence of driver genes in NSCLC, and whether they may have different tumor features affecting immunotherapy is still unclarified.

**Methods:**

Genomic alterations in 14 lung cancer‐related genes were conducted in 3440 Chinese NSCLC patients using next‐generation sequencing. Meanwhile, tumor mutational burden and immunotherapy dataset from the Memorial sloan kettering cancer center (MSKCC) and lung adenocarcinoma dataset from The Cancer Genome Atlas (TCGA) were utilized for analyzing the impact of the co‐occurring alterations on patients’ survival following immunotherapy.

**Results:**

In this cohort, 90.17% of patients had at least one somatic alteration in the 14 genes, including 51% of co‐occurring alterations. TP53 and epidermal growth factor receptor (EGFR) were the most prevalent genes (54.74% and 53.55%, respectively), followed by *KRAS*, *ERBB2*, *ALK*, *PIK3CA*, *ROS1*, *RET*, *MET*, *BRAF*, *KIT*, *FGFR1*, *PDGFRA*, and *NRAS*. The prevalence of *TP53*, *EGFR*, and *ERBB2* in our cohort were significantly higher than that from the TCGA database, whereas *KRAS*, *BRAF*, and *PDGFRA* were significantly lower than the latter. Furthermore, the patients who harbored multiple alterations (8.86%, 31/350) in eight driver genes survived longer and have a higher tumor mutation burden compared to the patients with a single alteration. Similar result was found between the patients with co‐occurring alteration of *EGFR* and other driver genes and the patients with single *EGFR* alteration. Meanwhile, we found a distinct immune cell infiltration feature between patients with single and multiple driver gene alterations, as well as between patients with only *EGFR* alteration and co‐occurring groups.

**Conclusion:**

This study identified a unique driver gene feature and found patients harboring co‐occurring alterations of *EGFR* and other driver genes may benefit from immunotherapy, which may provide more therapeutic selections for EGFR‐mutated NSCLC patients and merit additional investigation.

## Graphical abstract

This study identified a unique driver gene feature and found patients harboring co‐occurring alterations of epidermal growth factor receptor (EGFR) and other driver genes may benefit from immunotherapy, which may provide more therapeutic selections for EGFR‐mutated non‐small cell lung cancer patients and merit additional investigation.

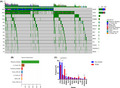

## 1 INTRODUCTION

Lung cancer is the leading cause of cancer‐related mortality worldwide, causing over 1.7 million deaths annually.^1^ Non‐small cell lung cancer (NSCLC) accounts for 85% of lung cancer cases.^2^

With the discovery of cancer driver genes, genomic testing has been integrated as a part of the standard diagnostic procedure, and several molecular drugs targeting the driver genes have been applied in the treatment of lung cancer and have shown great effectiveness in increasing the survival of advanced NSCLC.^3^, ^4^ Epidermal growth factor receptor (*EGFR*) alterations, including L858R and short insertions/deletions (indels) in exon 19, were identified as the first druggable alterations in NSCLC and proved to be the most robust predictive biomarker for *EGFR* tyrosine kinase inhibitors (TKIs).^5^ Since then, several additional driver gene alterations have been reported, including oncogenic somatic alterations in *BRAF*,^6^ intragenic insertions in *ERBB2* (in exon‐20),^7^ exon 14 skipping alterations in the *MET* proto‐oncogene,^8^ oncogenic alterations in *KRAS*,^9^ and genes rearrangement of *ALK*, *ROS1*, and *RET*.^10^ The National Comprehensive Cancer Network (NCCN) guideline recommends broad molecular profiling, including screening for the presence of activating alterations in *EGFR*, *ALK*, *ROS1*, *BRAF*, *KRAS*, *MET*, *ERBB2*, and *RET* to inform the selection of effective targeted therapies for NSCLC patients. Additionally, *TP53*, *PIK3CA*, *KIT*, *FGFR1*, *PDGFRA*, or *NRAS* were previously identified prevalent alterations in patients with NSCLC, and their impacts on target treatment or prognosis have received widespread attention.^11^, ^12^, ^13^ All of the 14 genes mentioned above can be considered lung cancer‐associated genes.

Immunotherapy is considered as a salvage treatment for patients with actionable driver alterations after the progression of related targeted therapies and chemotherapy.^14^ However, most clinical trials have shown that immune checkpoint inhibitors (ICIs) have poor activity in patients with driver gene alteration, especially *EGFR* and *ALK*. One retrospective study for advanced NSCLC patients with at least one oncogenic driver alteration receiving ICI monotherapy found that the median progression‐free survival (PFS) was only 2.8 months, and the objective response rates by driver alteration were generally low except *RET* (6%) and *ALK* (0%).^15^ Thus, therapeutic options are restrained in NSCLC patients with driver gene alterations, which is an urgent issue that needs to be addressed.

Recently, studies have found the presence of driver genes' co‐occurring alterations in NSCLC, and its effect on molecularly targeted therapies has attracted focus.^16^ Multiple clinical studies have found patients with co‐occurring alterations of *TP53* and *EGFR* alterations had worse prognostic when treated with *EGFR*‐TKI therapy.^16^ Besides, Martín Martorell et al. found that targeted treatment might not be as effective in patients with coexisting of *EGFR*, *KRAS*, *BRAF* alterations, and *ALK* rearrangement.^17^ However, the effect of the co‐existence of driver genes in NSCLC on immunotherapy is still unclarified.

In the present study, genomic alterations of 14 lung cancer‐associated genes were assessed in a cohort of 3440 Chinese NSCLC patients by next‐generation sequencing (NGS). The basic profile of the patient's driver gene alterations was described and compared with corresponding data in The Cancer Genome Atlas (TCGA) to better understand driver gene features in Chinese NSCLC patients. Furthermore, we focused on the patterns of co‐existence of driver genes and their effects on the response to immunotherapy.

## 2 MATERIALS AND METHODS

### 2.1 DNA isolation

The formalin fixation and paraffin‐embedding (FFPE) samples and fresh‐frozen tissues were collected and used for gDNA isolation. The specimens selected contained more than 20% tumor cells. The purified gDNA was quantified using the Qubit 3.0 Fluorometer (Life Technologies, Inc.) and StepOnePlus System (Life Technologies, Inc.).

### 2.2 Target NGS

Hundred nanograms of gDNA were sheared to target 200 bp fragment sizes with a Covaris E210 system (Covaris, Inc.). NGS of tumor gDNA was performed, in which Accel‐NGS 2S DNA Library Kit (Swift Biosciences, Inc.) was used for library preparation and xGen Lockdown Probes Kit (IDT, Inc.) for target enrichment. The custom xGen Lockdown probe was synthesized by IDT, Inc. for the exons and the part of introns of 14 genes of interest (*EGFR*, *ALK*, *ROS1*, *TP53*, *ERBB2*, *BRAF*, *KRAS*, *MET*, *PIK3CA*, *NRAS*, *FGFR1*, *RET*, *KIT*, and *PDGFRA*).

The prepared library was quantified by the Qubit 3.0 Fluorometer (Life Technologies, Inc.), and quality and fragment size were measured with an Agilent 2100 Bioanalyzer (Agilent Technologies, Inc.).

The samples underwent paired‐end sequencing on an Illumina NextSeq CN500 platform (Illumina, Inc.) with a 150 bp read length. Mean coverage beyond 1300× was achieved for tumor gDNA.

### 2.3 Data processing

Raw sequencing data were aligned to the reference human genome (UCSC hg19) through Burrows–Wheeler Aligner.^18^ After the duplicate removal and local realignment, the Genome Analysis ToolKit (GATK) v3.7 was used for single nucleotide variation (SNV)/indel calling and filtering.^19^ Gene fusions were called using Genefuse v0.6.0.^20^ The somatic variants were generated for the patient by subtracting the germline variants from the tumor to keep only variants unique to a tumor. The variants were annotated using the ANNOVAR software tool.^21^ The somatic alterations were annotated with information from the Catalog of Somatic Alterations in the OncoKB database.

### 2.4 Data sources

Tumor mutational burden (TMB) and Immunotherapy (MSKCC, Nat Genet 2019) dataset^22^ and clinical data were downloaded from cBioPortal (https://www.cbioportal.org), which contains 350 NSCLC samples in total, and all samples with alteration data were selected for alteration and survival analysis. Besides, lung adenocarcinoma (LUAD; TCGA, Firehose Legacy) dataset and mRNA expression data were downloaded from cBioPortal to compare the differences of immune microenvironment between patients with single *EGFR* alteration and the co‐existing alterations of *EGFR* and other driver genes.

### 2.5 Statistical analysis

Statistical analyses were performed using SPSS, GraphPad Prism 7 software, and R language statistical package. The differences between the two groups were assessed using Student's *t*‐test. The differences were considered significant if *p* < 0.05. The adjusted odds ratios were calculated. A two‐sided *p*‐value of <0.05 was considered to be statistically significant if there was no alpha correction. The overall survival (OS) curves were constructed using the Kaplan–Meier method, and the log‐rank test was performed. A *p* value <0.05 was considered to be statistically significant unless additionally specified.

## 3 RESULTS

### 3.1 Samples and clinical data description

A total of 2833 FFPE samples and 607 fresh‐frozen tissues were collected from 3440 patients diagnosed with NSCLC. Adenocarcinoma was the common histological type in this cohort, accounting for 92.7% (3189). Of the total 3440 patients, 1856 were male (53.95%), and 1584 were female (46.05%). The age at diagnosis ranged from 19 to 98 years old, with a median of 62 years (Table 1).

**TABLE 1:** Clinical characteristics of 3440 NSCLC patients

Characteristics	Total, *n* = 3440
Median age (range)	62 (19–98)
Gender	
Male	1856
Female	1584
Histology	
Adenocarcinoma	3189
Squamous	217
Adenosquamous	23
Large cell	11
Stage	
II–III	1760
IV	1680
Smoking history	
Yes	1430
No	1839
NA	171

Abbreviation: NSCLC, non‐small cell lung cancer; NA: no data.

### 3.2 Landscape of genomic alterations in 3440 NSCLC patients

Utilizing targeted deep sequencing of all exons and selected introns of 14 lung cancer‐related genes in 3440 NSCLC tissue samples, we found that 90.17% (3102 out of 3440) of patients had at least one somatic alteration. Among the 14 genes, 39.16% (1347/3440) of NSCLC patients were found to have a single alteration. 51.02% (1755/3440) harbored multiple alterations: 36.02% (1239/3440) had double alterations, 12.21% (420/3440) had triple alterations, and 2.79% (96/3440) had more than three alterations (Table S1).

**FIGURE 1:**
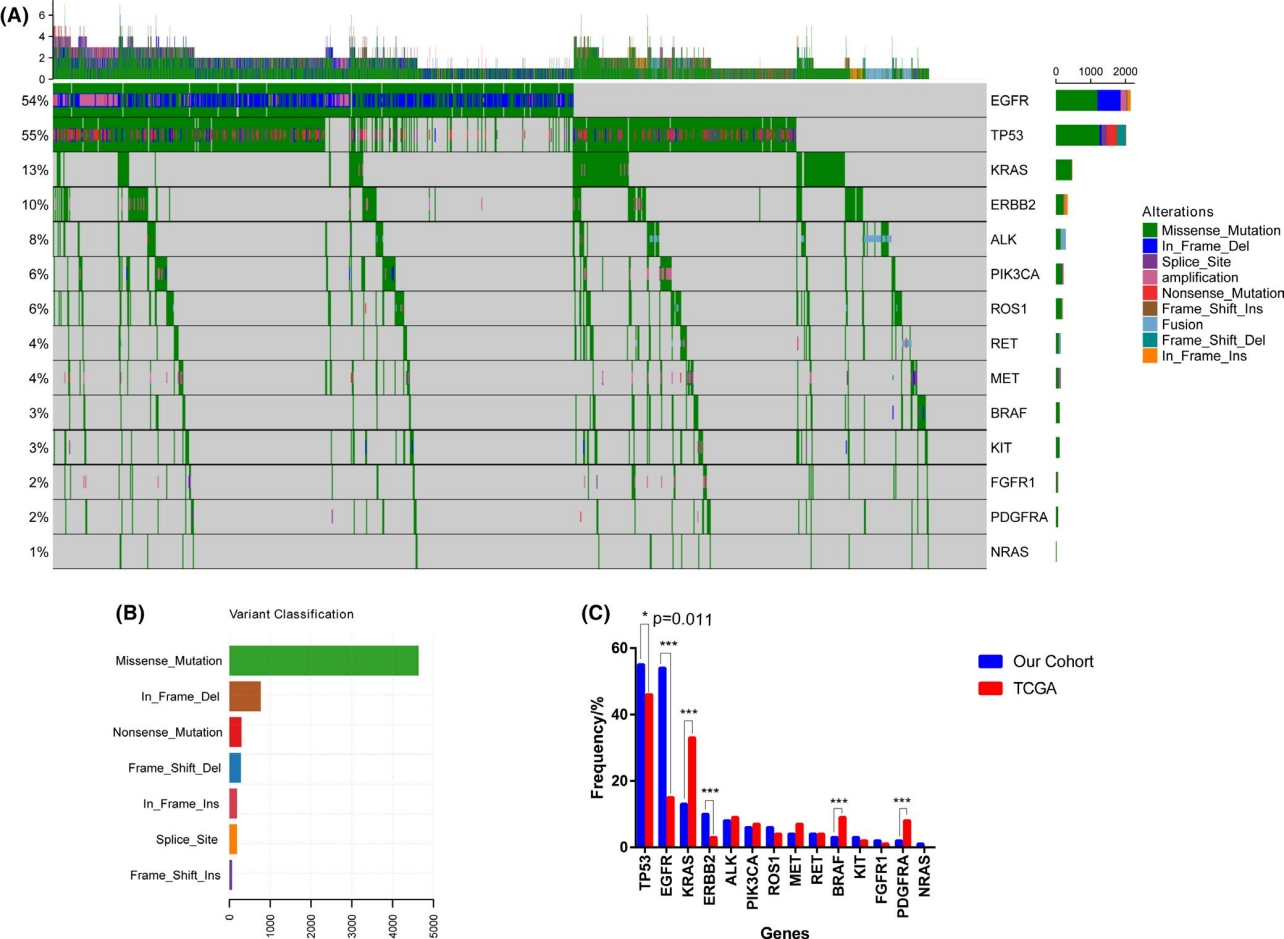
Landscape of somatic alterations in NSCLC involved in this study. (A). The landscape of alteration in 3440 NSCLC patients (B). Variant classification of all alterations (C). Comparison of the alteration frequencies of the 14 cancer‐related genes between our cohort and the TCGA cohort. Two‐sided Fisher's tests were conducted to compare the different frequencies between two cohorts. ^***^ *p* ≤ 0.001, *p ≤ 0.05. NSCLC, non‐small cell lung cancer; TCGA, The Cancer Genome Atlas

In this study, the most prevalent genes were *TP53* (54.74%), *EGFR* (53.55%), and *KRAS* (13.40%) (Figure 1A), followed by *ERBB2* (9.51%), *ALK* (7.82%), *PIK3CA* (6.34%), *ROS1* (5.78%), *RET* (4.01%), *MET* (3.92%), *BRAF* (3.14%), *KIT* (3.05%), *FGFR1* (1.98%), *PDGFRA* (1.86%), and *NRAS* (0.55%). Among the 14 genes in our cohort, except for *PIK3CA* (6.3% vs. 12.0%) and *FGFR1* (1.98% vs. no data), the prevalence of the other 12 genes was similar to the results reported in a previous Chinese NSCLC population.^11^ The variant classification spectrum showed that missense alteration type was the most common, followed by frameshift deletion and nonsense alteration (Figure 1B). Comparing the prevalence of 14 genes in the LUAD patients from the TCGA database identified significant differences in *TP53* (54.74% vs. 46.09%), *EGFR* (53.55% vs. 14.35%), *KRAS* (13.40% vs. 32.61%), *ERBB2* (9.51% vs. 2.61%), *BRAF* (3.14% vs. 9.57%), and *PDGFRA* (1.86% vs. 6.09%) in our cohort (Figure 1C). Furthermore, the prevalence of 14 genes in both LUAD and lung squamous cell carcinoma (LUSC) patients was calculated and compared (Figure S1). We found that the alteration of *EGFR*, *KRAS*, and *ALK* occurred more often in patients with LUAD than LUSC (*p* < 0.05), however, the frequencies of *TP53*, *PIK3CA*, and *FGFR1* were significantly lower than the latter (*p* < 0.01), which was similar to the previous report.^23^

### 3.3 Alteration analysis of 14 genes

**FIGURE 2:**
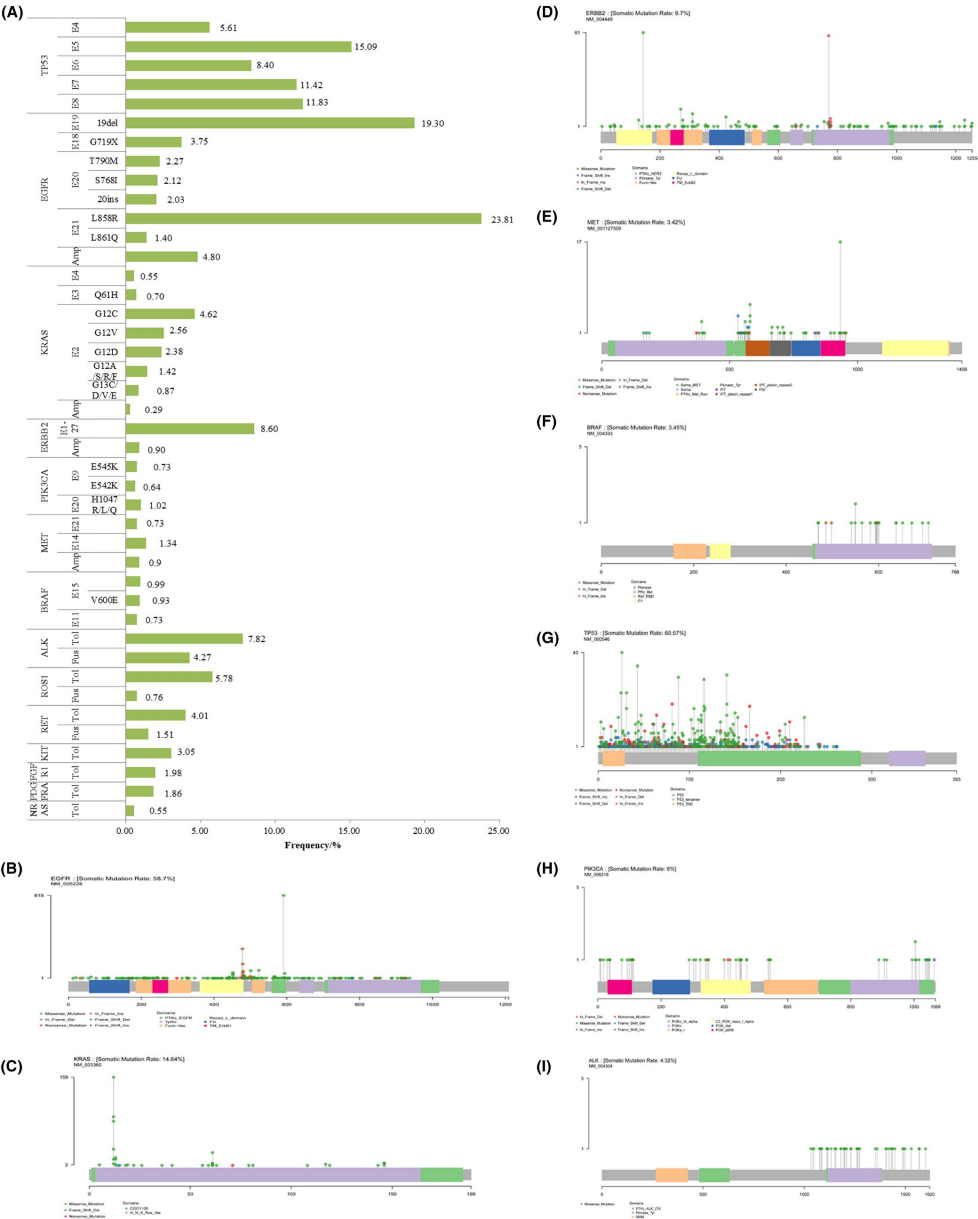
Somatic alteration frequencies of 14 cancer‐related genes (A). The main alteration sites and frequency of 14 genes. The lollipop plot shows the genomic distribution of *EGFR* (B), *KRAS* (C), *ERBB2* (D), *MET* (E), *BRAF* (F), *TP53* (G) *PIK3CA* (H), and *ALK* (I). The gray bar represents the entire protein with the different amino acid positions. The length of the gray lines indicates the number of alterations detected at the specified position, and the colored circles on the gray bar represent the corresponding alteration types. The colored boxes are different functional domains. Amp, amplification; Fus, Fusion; Tol, Total alteration frequency; EGFR, epidermal growth factor receptor

In this study, 53.55% of NSCLC patients in our cohort had *EGFR* alternations, most of which had been well developed as actionable variants, such as L858R, exon 19 del, exon 20‐ins, L861Q, G719X, S768I, and T790 M (Figure 2A). Besides, 112 rare *EGFR* alterations were found in the cohort (Table S2). As profiled in Figure 2B, alterations of *EGFR* are distributed relatively throughout the whole protein. Multiple *EGFR* alterations were found in 18.72% of patients (644/3440). Of the 644 patients, 78.73%, 15.37%, and 5.12% of them had double alterations, triple alterations, and quadruple alterations, respectively. *KRAS* alteration was detected in 13.40% of patients (461/3440), and most of the alterations were located in exon 2 (11.66%, 401/3440); the remaining ones were detected in exon 3 (1.16%, 40 out of 3440) and exon 4 (0.55%, 19 out of 3440). The most prevalent alterations included G12C (4.62%), G12D (2.38%), and G12V (2.56%) (Figure 2A,C). *ERBB2* alterations were detected in 327 patients (9.51%), distributed throughout the whole protein, of which a quarter (26.00%, 85/327) located in exon 20. The *ERBB2*‐positive cases featured samples with more nonsynonymous SNV (6.40%, 220/3440), nonframeshift insertion (2.33%, 80/3440), and amplification (0.90%, 31/3440) (Figure 2A,D). A total of 135 (3.92%, 135/3440) *MET* alterations were detected, of which 46 (1.34%, 46/3440) located in exon 14, 25 (0.73%, 25/3440) exon 21, 31 (0.9%, 31/3440) other location, and 31 (0.9%, 31/3440) amplifications (Figure 2A,E). We identified 3.14% of patients (108/3440) harbor *BRAF* alteration, and 0.93% (32/3440) were V600E. Most of the remaining were located in exon 15 (0.99%, 34/108) and exon 11 (0.73%, 25/108) (Figure 2A,F). *TP53* was the most frequently mutated gene, detected in 54.74% of patients (1883/3440), which contains more nonsense alteration. The sites of *TP53* alteration were mostly located in exon 5–8 (46.74%, 1608/3440) (Figure 2A,G). *PIK3CA* alteration was detected in 6.34% of patients (218/3440), of which 1.60% were located in exon 9 (55/3440) and exon 20 (1.83%, 63 /3440). There were 25 (0.73%, 25/3440) cases of E545K, 35 (1.02%, 35/3440) cases of H1047R/L/Q, and 22 cases (0.64%, 22/3440) of E542K (Figure 2A,H).

Other genomic alterations were as follows: *ALK* (7.82%, 269/3440), *ROS1* (5.78%, 199/3440), *RET* (4.01%, 138/3440), *KIT* (3.05%, 105/3440), *FGFR1* (1.98%, 68/3440), *PDGFRA* (1.86%, 64/3440), and *NRAS* (0.55%, 19/3440) (Figure 2A,I; Figure S2).

### 3.4 *ALK*, *ROS1*, and *RET* fusions in NSCLC

**FIGURE 3:**
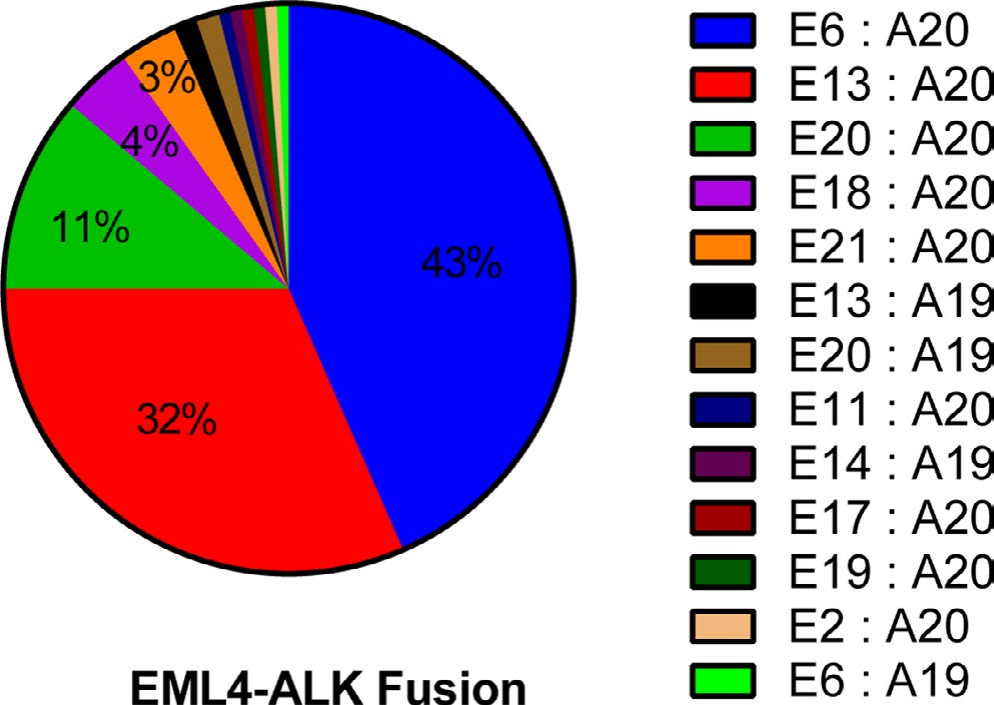
The frequency and distribution of *EML4*‐*ALK* fusion subtypes identified in the NSCLC cohort. NSCLC, non‐small cell lung cancer

In the cohort, 147 patients (4.27%) had *ALK* rearrangements, of which 97.28% (143/147) were *EML4*‐*ALK*, and 4 other *ALK* fusions (2 *CLIP1*‐*ALK*, 1 *HIP1*‐*ALK*, and 1 *KIF5B*‐*ALK*). The frequency of *EML4*‐*ALK* subtypes is shown in Figure 3 as the most common subtypes of *EML4*‐*ALK* were E6:A20 (variant 3; 43.42%) and E13:A20 (variant 1; 31.58%), whereas E20:A20 (variant 2) accounted for 11.18% (Figure 3).

We also find 1.51% (52/3440) of patients have *RET* rearrangement (36 *KIF5B*‐*RET*, 12 *CCDC6*‐*RET*, *2 ERC1*‐*RET*, and 2 *NCOA4*‐*RET*) and 0.76% (26/3440) of patients harbor *ROS1* rearrangement (14 *CD74*‐*ROS1*, 5 *EZR*‐*ROS1*, 2 *LRIG3*‐*ROS1*, 2 *SLC34A2*‐*ROS1*, 2 *SDC4*‐*ROS1*, and 1 *ERC1*‐*ROS1*) in this cohort (Figure 2A).

### 3.5 Patients’ characteristics and somatic alterations

We evaluated the association between alteration in 14 genes and gender and found that the alteration rate of *EGFR* (male vs. female: 41.47% vs. 67.91%, *p* < 0.0001) and *ALK* (male vs. female: 6.59% vs. 9.30%, *p* = 0.003) were higher in female than in male patients in our cohort. Whereas, significantly higher prevalence of the *TP53* (63.12% vs. 44.94%, *p* < 0.0001), *KRAS* (17.17% vs. 8.92%, *p* < 0.0001), *KIT* (3.62% vs. 2.28%, *p* = 0.027), and *FGFR1* (2.43% vs. 1.46%, *p* = 0.049) was found in male patients. There was no significant difference between male and female NSCLC patients for the alterations rates of other genes (*ERBB2*, *PIK3CA*, *ROS1*, *RET*, *MET*, *BRAF*, *PDGFRA*, and *NRAS*). Similar to *EGFR* alteration, the rearrangements of *ALK* (male vs. female: 3.40% vs. 5.32%, *p* = 0.007) and *ROS1* (male vs. female: 0.43% vs. 1.14%, *p* = 0.028) were enriched in females patients. Besides, we found the median age in the *ALK* (median age 57, range 31–84) and *ROS1* (median age 58.5, range 30–76) rearrangements‐positive cohort was lower than the whole cohort (median age 62, range 19–98), which demonstrates that younger patients were more likely to harbor *ALK* and *ROS1* rearrangements.

### 3.6 Co‐occurring alterations of driver gene in NSCLC

**FIGURE 4:**
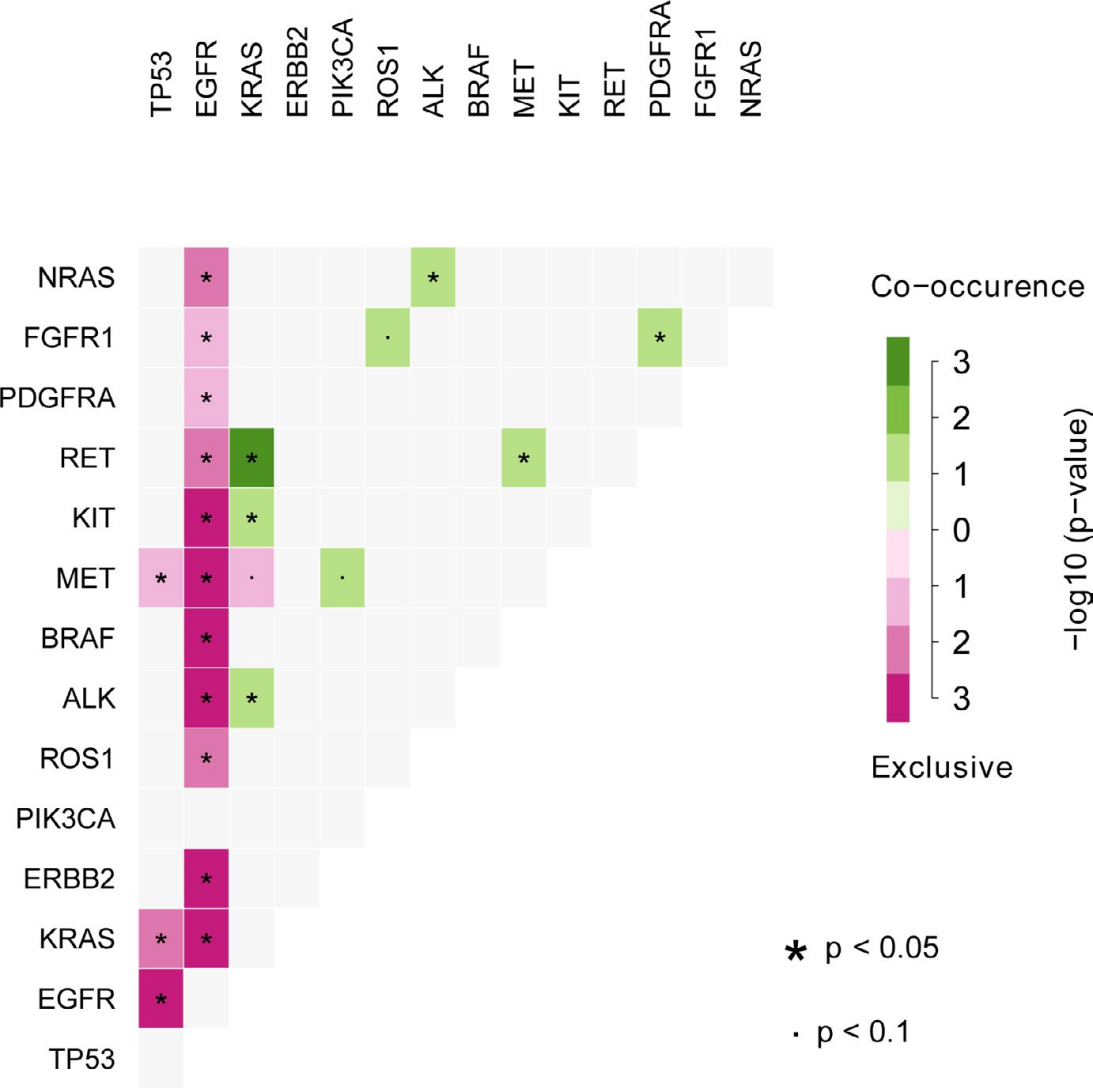
Co‐occurrence or exclusivity of 14 genes alterations events in the NSCLC (*n* = 3440). The green box represents the pair genes are significantly co‐altered, whereas the purple box indicates the two genes are significantly mutually exclusive. The depth of the color reflects the size of the *p* value that the darker the color, the smaller the *p* value. NSCLC, non‐small cell lung cancer

The frequencies of co‐occurring alterations in 14 cancer‐related genes were identified as 51.02% (1755/3440) in our cohort (Table S1). The more common genes co‐occurring with *EGFR* were *TP53* (28.26%), *ERBB2* (3.66%), *PIK3CA* (3.14%), *ROS1* (2.38%), and *KRAS* (28.26%). Besides, 7.15% of patients carried co‐occurring alterations of *KRAS* and *TP53*. Mutually exclusive or co‐occurring set of 14 genes were detected using the somatic interactions function of the maftools package, which performs pair‐wise Fisher's Exact test to detect such significant pair of genes. As a result, six pairs of significantly co‐altered genes were found in the study, including the co‐occurring in *KRAS* and *RET*/*KIT*/*ALK*; *NRAS* and *ALK*; *MET* and *RET*; *PDGFRA* and *FGFR1*. It is worth noting that *EGFR* alterations were mutually exclusive with the other 12 gene alterations except for *PIK3CA* (Figure 4). Besides, *TP53* was mutually exclusive with *MET*/*KRAS*.

Eight driver genes, *EGFR*, *ROS1*, *MET*, *RET*, *ALK*, *ERBB2*, *KRAS*, and *BRAF*, are recommended by the NCCN guideline to inform the selection of effective targeted therapies for NSCLC patients. For the eight driver genes, approximately 80.87% (2782/3440) of Chinese NSCLC patients harbored at least one alteration, and single and multiple alterations (co‐occurring alterations) were accounted for 77.89% (2167/2782) and 22.11% (615/2782), respectively (Table 2). To find the profile (frequency) of Western populations, we calculated that in the corresponding TCGA (*n* = 230) and the MSKCC (*n* = 350) datasets. As shown in Table 2, fewer patients of Western populations (our cohort vs. TCGA: 80.87% vs. 65.65%, *p* < 0.0001; our cohort vs. MSKCC: 80.87% vs. 62.57%, *p* < 0.0001) carried alteration in eight driver genes compared with our cohort. Among the eight driver genes, *EGFR* and *KRAS* were the more common alterations, and the co‐occurring alterations, including *EGFR* or *KRAS,* have attracted wide attention. Thus, patients from the three cohorts were divided into eight groups according to the type and number of the altered gene they carried, including *EGFR*/*KRAS*_S (Patients with single *EGFR*/*KRAS* alteration), *EGFR*/*KRAS*_M (Patients with co‐occurring alterations of *EGFR*/*KRAS* and other seven driver genes), ALL/Others_S [Patients with single alteration in eight driver genes/others six driver genes (*ROS1*, *MET*, *RET*, *ALK*, *ERBB2*, *BRAF*)], and ALL/Others_M (Patients with multiple alterations in eight driver genes/other six driver genes) (Table 2).

**TABLE 2:** Comparison of co‐occurring alterations in Chinese, TCGA, and MSKCC cohorts

Groups	Chinese (*n* = 3440)	TCGA (*n* = 230)	*p* value Chinese versus TCGA	MSKCC (*n* = 350)	*p* value Chinese versus MSKCC
Positive	80.87% (2782/3440)	65.65% (151/230)	<0.0001	62.57% (219/350)	<0.0001
Negative	19.13% (658/3440)	34.35% (79/230)		37.43% (131/350)	
ALL_S	77.89% (2167/2782)	76.82% (116/151)	0.763	85.84% (188/219)	0.005
ALL_M	22.11% (615/2782)	23.18% (35/151)		14.16% (31/219)	
*EGFR*_S	50.86% (1415/2782)	17.88% (27/151)	0.839	16.89% (37/219)	0.199
*EGFR*_M	15.35% (427/2782)	4.64% (7/151)		2.74% (6/219)	
*KRAS*_S	9.49% (264/2782)	33.77% (51/151)	0.054	46.58% (102/219)	<0.0001
*KRAS*_M	7.08% (197/2782)	14.57% (22/151)		10.05% (22/219)	
Others_S	17.54% (488/2782)	25.17% (38/151)	0.227	22.37% (49/219)	0.641
Others_M	2.19%(61/2782)	5.30% (8/151)		1.83% (4/219)	

Note

Positive, At least one alteration in eight driver genes (*EGFR*, *KRA*S, *ROS1*, *MET*, *RET*, *ALK*, *ERBB2*, and *BRAF*); Negative, Non alteration in eight driver genes; ALL/Others_S, Single alteration in eight driver genes/others six driver genes (*ROS1*, *MET*, *RET*, *ALK*, *ERBB2*, and *BRAF)*; ALL/Others_M, Multiple alterations in eight driver genes/other six driver genes; *EGFR*/*KRAS*_S, Single *EGFR*/*KRAS* alteration; *EGFR*_M, Co‐occurring alterations of *EGFR* and other seven driver genes; *KRAS*_M, Co‐occurring alterations of *KRAS* and other seven driver genes.

Abbreviations: EGFR, epidermal growth factor receptor; TCGA, The Cancer Genome Atlas; MSKCC, Memorial Sloan Kettering Cancer Center.

### 3.7 Patients with multiple alterations have a longer survival time and higher TMB score

**FIGURE 5:**
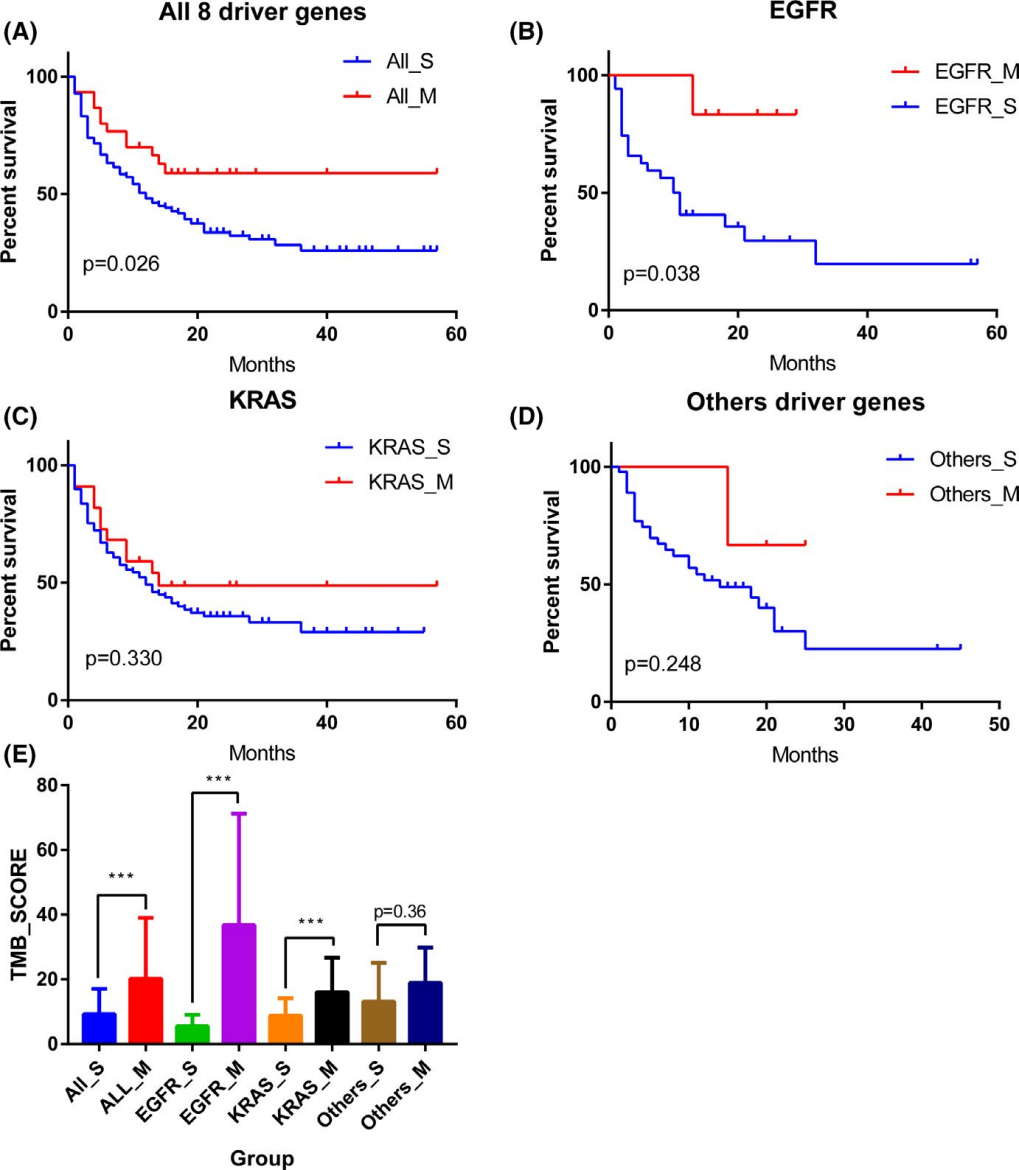
Multiple alterations are associated with longer survival and higher TMB score in patients after immunotherapy (A). Patients with multiple alterations in eight driver genes (ALL_M Group) (*EGFR*, *ROS1*, *MET*, *RET*, *ALK*, *ERBB2*, *KRAS*, and *BRAF*) have better overall survival (*p* = 0.026) (B). Patients with co‐occurring alterations of *EGFR* and the other seven driver genes (*EGFR*_M Group) have better overall survival (*p* = 0.038) (C). Patients with co‐occurring alterations of *KRAS* and other seven driver genes (*KRAS*_M group) have better overall survival (*p* = 0.330) (D). Patients with multiple alterations in the other six driver genes (Others_M Group) (*ROS1*, *MET*, *RET*, *ALK*, *ERBB2*, and *BRAF*) have better overall survival (*p* = 0.248) (E). Patients with multiple alterations (ALL_M, *EGFR*_M, *KRAS*_M, and Others_M Group) have higher TMB levels. ^***^ *p* ≤ 0.001; EGFR, epidermal growth factor receptor; TMB, tumor mutational burden

To study the effects of the co‐existing driver alterations on the survival of immunotherapy, we compared the difference in survival between patients with single alteration (ALL_S group) and patients with multiple alterations (ALL_M group) in the MSKCC cohort and found an interesting result that the latter has a significantly longer survival time (median survival: 12 months vs. unreach, *p* = 0.026) (Figure 5A). Similarly, group *EGFR*_M survived significantly longer than group *EGFR*_S (median survival: unreach vs. 11 months; *p* = 0.038) (Figure 5B), group *KRAS*_M survived longer than group KRAS_S (median survival: 14 vs. 12 months; *p* = 0.330) (Figure 5C), and group Others_M survived longer than Others_S (median survival: unreach vs. 14 months; *p* = 0.248) (Figure 5D). In summary, among immunotherapy patients, those with multiple alterations in the eight driver genes have a longer survival time. Meanwhile, we revealed that the patients with multiple alterations in eight driver genes had higher TMB levels (Figure 5E; Table S3).

All 350 NSCLC patients in the MSKCC dataset received PD‐1/PD‐L1‐targeted monotherapy (atezolizumab, durvalumab, nivolumab, or pembrolizumab) or combination immunotherapy (Combo; 6%, 21/350).^22^ Interestingly, the patients who received the combination therapy had better survival than those who were treated with PD‐1/PD‐L1‐targeted monotherapy (median survival: 46 vs. 11 months; *p* = 0.009) (Figure S3). However, Fisher's exact test results proved that the difference in survival between patients with single alteration (All_S/*EGFR*_S) and those with multiple alterations (All_M/*EGFR*_M) was independent of the drug class of patients received (all *p* > 0.05, Table S4).

### 3.8 The differences of immune microenvironment between patients with single and multiple alterations

**FIGURE 6:**
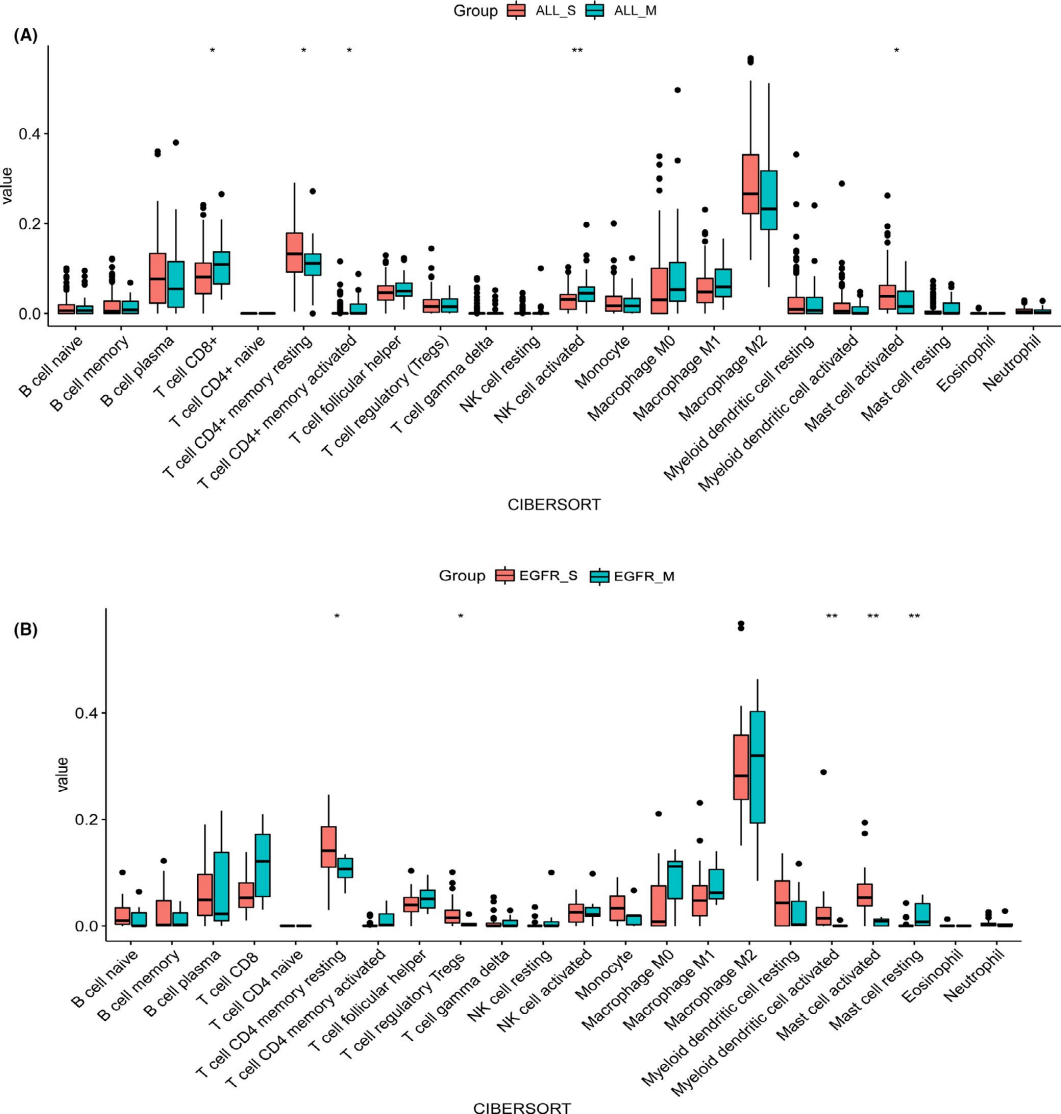
Tumor‐infiltrated immune cells in NSCLC patients with co‐occurring alterations from the TCGA cohort. (A) Patients with single driver gene alteration (ALL_S) versus co‐occurring alterations in eight driver genes (ALL_M). (B) The patients with only *EGFR* (*EGFR*_S) versus co‐occurring alterations of *EGFR* and other seven driver genes (*EGFR*_M). *p* < 0.05 for all eligible samples. ^**^ *p* ≤ 0.01, ^*^ *p* ≤ 0.05. EGFR, epidermal growth factor receptor; NSCLC, non‐small cell lung cancer; TCGA, The Cancer Genome Atlas

To find why patients with multiple alterations (the ALL_M and *EGFR*_M group) had better survival outcomes compared to the patients with single *EGFR* alteration (the ALL_S and the *EGFR*_S group) after immunotherapy, we investigated the fractions of tumor‐infiltrated immune cells (TIICs) between these groups in the TCGA cohort. The expression signature matrix of the 22 infiltrated immune cell types was analyzed based on CIBERSORT software. M2 macrophages accounted for a large proportion of NSCLC immune cell infiltration both in the four groups (Figure 6A,B). The fractions of five TIICs varied significantly among ALL_S and ALL_M groups. Three TIICs (T cell CD8+, activated memory T cell CD4+, and activated natural killer [NK] cell) were in a higher proportion in the ALL_M group than those in the ALL_S group (*p* < 0.05), whereas resting memory CD4+ T cells and activated mast cells were in a higher proportion in the ALL_S group (*p* < 0.05). Similarly, resting memory T cell CD4+, regulatory T cell (Tregs), activated myeloid dendritic cell, and activated mast cells were more common in the *EGFR*_S group compared with the *EGFR*_M group (*p* < 0.05), and the *EGFR*_M group generally contained a higher fraction of resting mast cell than the *EGFR*_S group (*p* < 0.05). The results showed the heterogeneity of immune cell infiltration between patients with single and multiple alterations.

## 4 DISCUSSION

This study performed 14 cancer‐related gene alternation analyses in a lager Chinese NSCLC cohort (*n* = 3440), and identified 90.17% (3102/3440) of patients with at least one alteration, including *TP53* (54.74%), *EGFR* (53.55%), *KRAS* (13.40%), *ERBB2* (9.51%), *ALK* (7.82%), *PIK3CA* (6.34%), *ROS1* (5.78%), *RET* (4.01%), *MET* (3.92%), *BRAF* (3.14%), *KIT* (3.05%), *FGFR1* (1.98%), *PDGFRA* (1.86%), and *NRAS* (0.55%).

Previous studies have found that the alterations of driver genes are related to ethnicity. For example, in *KRAS*‐positive NSCLC, the patients in Western countries (about 25%) are much more than in Asia (10%–15%).^9^, ^24^, ^25^, ^26^ We also found this prevalence, that Chinese patients with NSCLC had a much higher frequency of *EGFR*, *ERBB2*, and *TP53* alterations but a significantly lower frequency of *KRAS*, *BRAF*, and *PDGFRA* alterations than the Western patient population. The alterations of *KRAS*, *KIT*, *FGFR1*, and *TP53* were significantly higher in males, while *EGFR* alterations and *ALK* rearrangement are more common in females. The genomic alterations profiling of Chinese NSCLC patients in this study was consistent with previous studies.^11^, ^27^

Non‐small cell lung cancer is the most commonly diagnosed cancer and the leading cause of cancer death. Fortunately, driver gene screening is widely use to guide molecular targeted therapy, which has shown great effectiveness in improved the prognosis. The patients of NSCLC with *EGFR* alteration may benefit from treatment using *EGFR* TKIs. In this study, 53.55% of NSCLC patients harbored *EGFR* alterations, and 43.11% of patients with *EGFR*‐L858R and exon 19 del alterations, which was consistent with another report.^11^ Less common alterations, such as L861Q, S768I, and G719X, accounted for approximately 7% of patients. Although these alterations are not sensitive to the *EGFR*‐TKI as same as L858R and exon 19 del, they had been proved to have a benefit from afatinib therapy.^28^

*KRAS* alterations are associated with a poor NSCLC prognosis. 13.40% of patients harbored *KRAS* alterations in this cohort, consistent with previous reports.^29^ *PIK3CA* plays a pivotal role in cell metabolism and proliferation and whose alterations are commonly found in a variety of cancers. 5.4% of patients harbored *PIK3CA* alterations in this cohort, and most of that is located in the helical binding domain (exon 9, E545K, or E542K) or the catalytic subunit (exon 20, H1047R, or H1047L), which are considered oncogenic and targetable.^30^, ^31^, ^32^, ^33^ *BRAF* alteration frequency is 3.14% in this cohort, 0.9% (22 out of 3440) harbored V600E alterations, which were significantly associated with shorter disease‐free and OS rates.^34^, ^35^ *TP53* gene was initially found to be essential for the DNA‐damage checkpoint, encodes a tumor suppressor protein (p53 protein) containing transcriptional activation, DNA binding, and oligomerization domains.^36^, ^37^ Most mutant p53 proteins have lost their DNA‐binding activity, leading to the loss of their growth inhibition and apoptotic properties.^38^ In this cohort, *TP53* (54.74%) was the most frequently altered gene and mainly on the DNA‐binding domain. Studies on primary East Asian patient populations have detected the *EML4*‐*ALK* fusion gene in 3%–7% of NSCLCs,^39^, ^40^, ^41^, ^42^ most commonly in adenocarcinomas and females. Similar to the previous studies, the incidence of *ALK* rearrangement was 4.27% in this cohort. Due to different breakpoints on *EML4*, several subtypes of the *EML4‐ALK* alteration have been described.^42^, ^43^, ^44^ The most common subtypes were E6:A20 (variant 3), E13:A20 (variant 1), and E20:A20 (variant 2), accounting for 43.42%, 31.58%, and 11.18% of all *EML4*‐*ALK* cases in our cohort, respectively. *EML4*‐*ALK* fusion serves as a therapeutic target for *ALK* TKIs and has shown promising results when treating NSCLC patients carrying *ALK* rearrangement.^45^ However, studies have suggested differential clinical responses to *ALK* inhibitors among different subtypes of *EML4*‐*ALK*. *EML4*‐*ALK* variant 3 may be a major source of *ALK* inhibitor resistance in the clinic. The stratification of patients with advanced *ALK* rearrangement‐positive NSCLC by the variant‐specific genotype should help to predict clinical responses to *ALK* inhibitors.^11^

There is mounting evidence that the presence of co‐occurring alterations in patients with NSCLC, analyzed the 3440 NSCLC Chinese patient cohort, we also identified 51.02% of NSCLC patients with co‐occurring alterations in 14 genes. Recently, some reports demonstrated that the presence of co‐occurring alterations presented challenges for NSCLC targeted therapy. For example, among *EGFR*‐altered NSCLC patients, *TP53* alterations reduce responsiveness to *EGFR*‐TKIs and worsen prognosis,^46^, ^47^ *KRAS* alteration was significantly associated with an absence of response to *EGFR*‐TKI,^48^ and *PIK3CA* alteration was associated with shorter OS in some studies but do not appear to impact response rates and PFS with first‐line or second‐line *EGFR*‐TKI therapy. Therefore, the *EGFR* alteration test alone may not be sufficient to determine a patient's sensitivity to TKI therapy. Among *EGFR*‐altered patients, the co‐occurring frequencies of *TP53*, *KRAS*, and *PIK3CA* were 28.26%, 2.15%, and 3.14%, respectively, and they may not benefit equally from *EGFR*‐TKI compared with patients with only *EGFR* alteration.

Many studies have shown that patients with *EGFR* alterations are unable to benefit from immunotherapy and that may be associated with the development of hyper progressive disease and lead to increased toxic effects.^49^, ^50^ Furthermore, previous studies have indicated that EGFR‐TKI might not be as effective in NSCLC patients with co‐occurring alterations of *EGFR* and other driver genes.^16^, ^17^ Thus, effective treatment is urgently needed for these NSCLC patients. Intriguingly, we found that NSCLC patients with co‐occurring alterations of *EGFR* and other driver genes have higher TMB levels and longer OS than patients with a single *EGFR* alteration after immunotherapy, and similar results were found between patients with multiple driver gene alterations and single alteration in eight driver genes. The results demonstrate that the coexistence of other gene alterations affects the effectiveness of immunotherapy, the underlying molecular mechanism of which needs further study. Meanwhile, we discovered that the fractions of TIICs varied among the *EGFR*_M and *EGFR*_S groups as well as between the ALL_M group and the ALL_S group. Patients harboring coexisting alterations of *EGFR* and other driver genes have lower fractions of resting memory CD4 T cell, regulatory T cell (Tregs), activated myeloid dendritic cell, and activated mast cell, and have higher fractions of resting mast cell. Previous studies found the differences in immune cell composition in NSCLC are associated with survival. For example, the higher fraction of resting mast cells is associated with longer survival time, but a higher fraction of active dendritic cells or activated tumor Tregs is correlated with a poor prognosis.^51^, ^52^ Cho et al. analyzed the immune cell composition in peripheral blood mononuclear cells from nine NSCLC patients pre‐ and post‐treatment with immunotherapy and found that NK cells were enriched in the immunotherapy responder group and with higher overall activity compared with that of non‐responders.^53^ In summary, the patients carried co‐occurring alterations of *EGFR* and other driver genes with longer survival and higher TMB score and had features of immune cell infiltration associated with better prognosis. Taken together, the patients with co‐occurring alterations of *EGFR* and other driver genes may benefit from immunotherapy, which may be associated with the immune microenvironment, and clinical research with a larger sample size is required to verify this result.

In conclusion, we performed NGS on a cohort of 3440 NSCLC patients to present a clear feature of driver gene alterations in Chines NSCLC patients. Besides, we identified that the co‐occurring of driver genes are associated with longer survival on immunotherapy. Importantly, patients harboring co‐occurring alterations of *EGFR* and other driver genes may benefit from immunotherapy, which may provide more therapeutic selections for *EGFR*‐mutated NSCLC patients and merit additional investigation.

## ETHICS STATEMENT

This study was approved by the ethics committee of Fifth Medical Center of Chinese PLA General Hospital and conducted under the principles of the Declaration of Helsinki and the Good Clinical Practice guidelines. All enrolled patients provided written informed consent.

## CONFLICT OF INTEREST

The authors declare that the research was conducted in the absence of any commercial or financial relationships that could be construed as a potential conflict of interest.

## Supporting information

Figure S1

Figure S2

Figure S3

Tables S1‐4

## Data Availability

The datasets analyzed for this study can be found in the cBioPortal [https://www.cbioportal.org]. The datasets used and/or analyzed during the current study are available from the corresponding author upon reasonable request.
