# Supplementary figures and images for: Driver gene alterations profiling of Chinese non‐small cell lung cancer and the effects of co‐occurring alterations on immunotherapy
Source: Cancer Med. 2021 Oct 2;10(20):7360–72. doi: 10.1002/cam4.4178 (PMC8525092; doi:10.1002/cam4.4178)

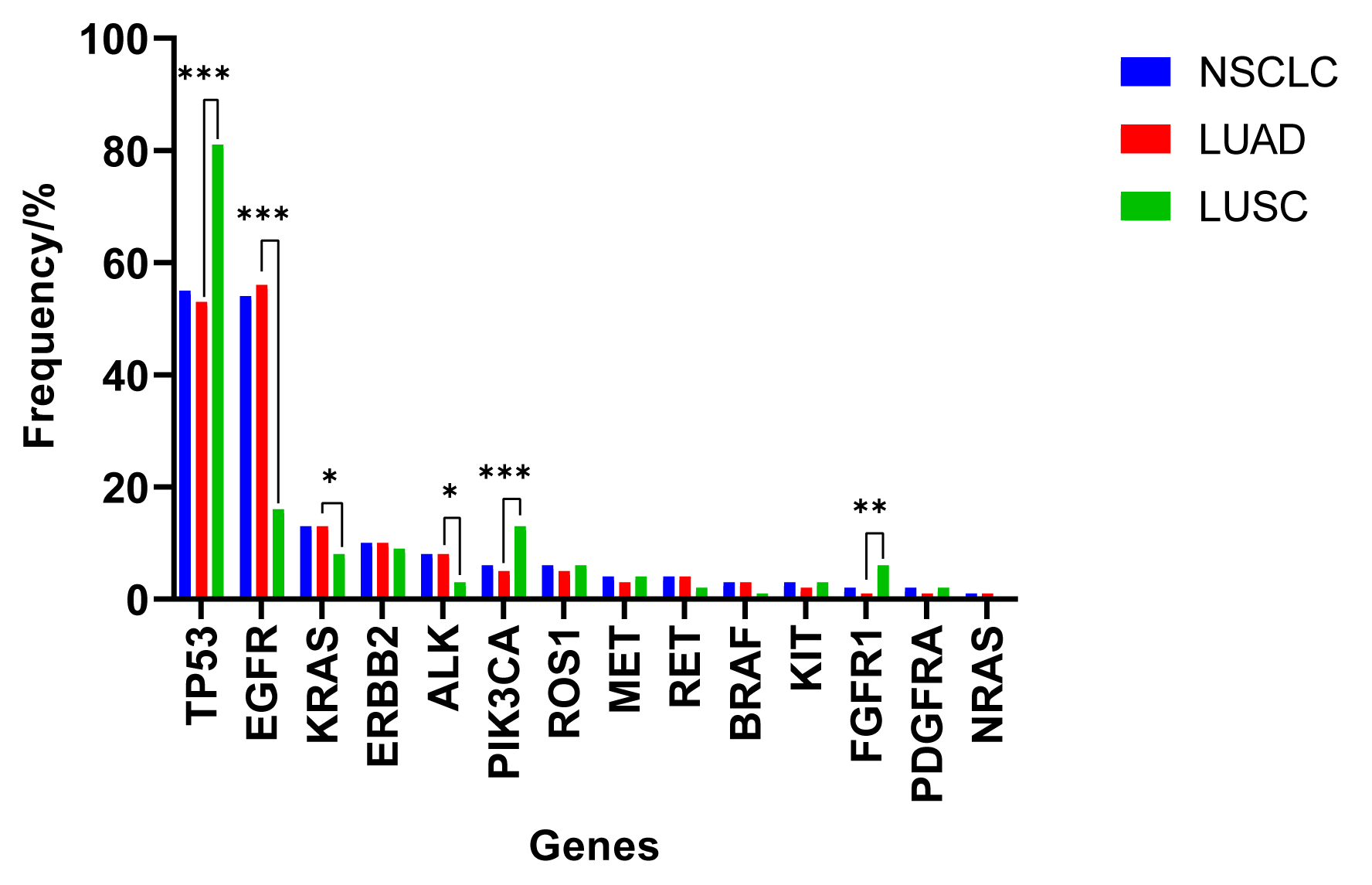

Supplement: Supplementary file 1 — Figure S1 [file CAM4-10-7360-s001.tif]

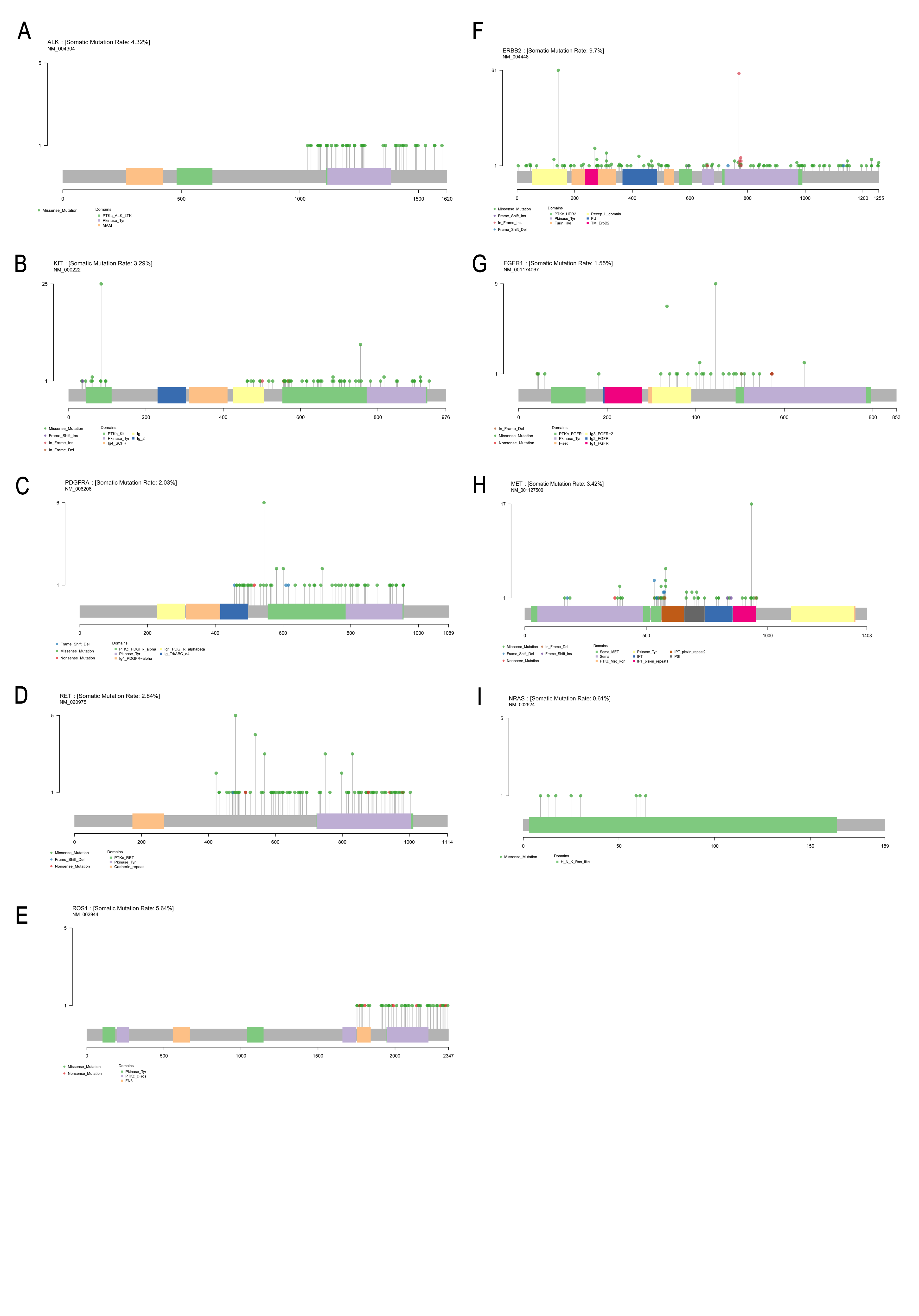

Supplement: Supplementary file 2 — Figure S2 [file CAM4-10-7360-s003.tif]

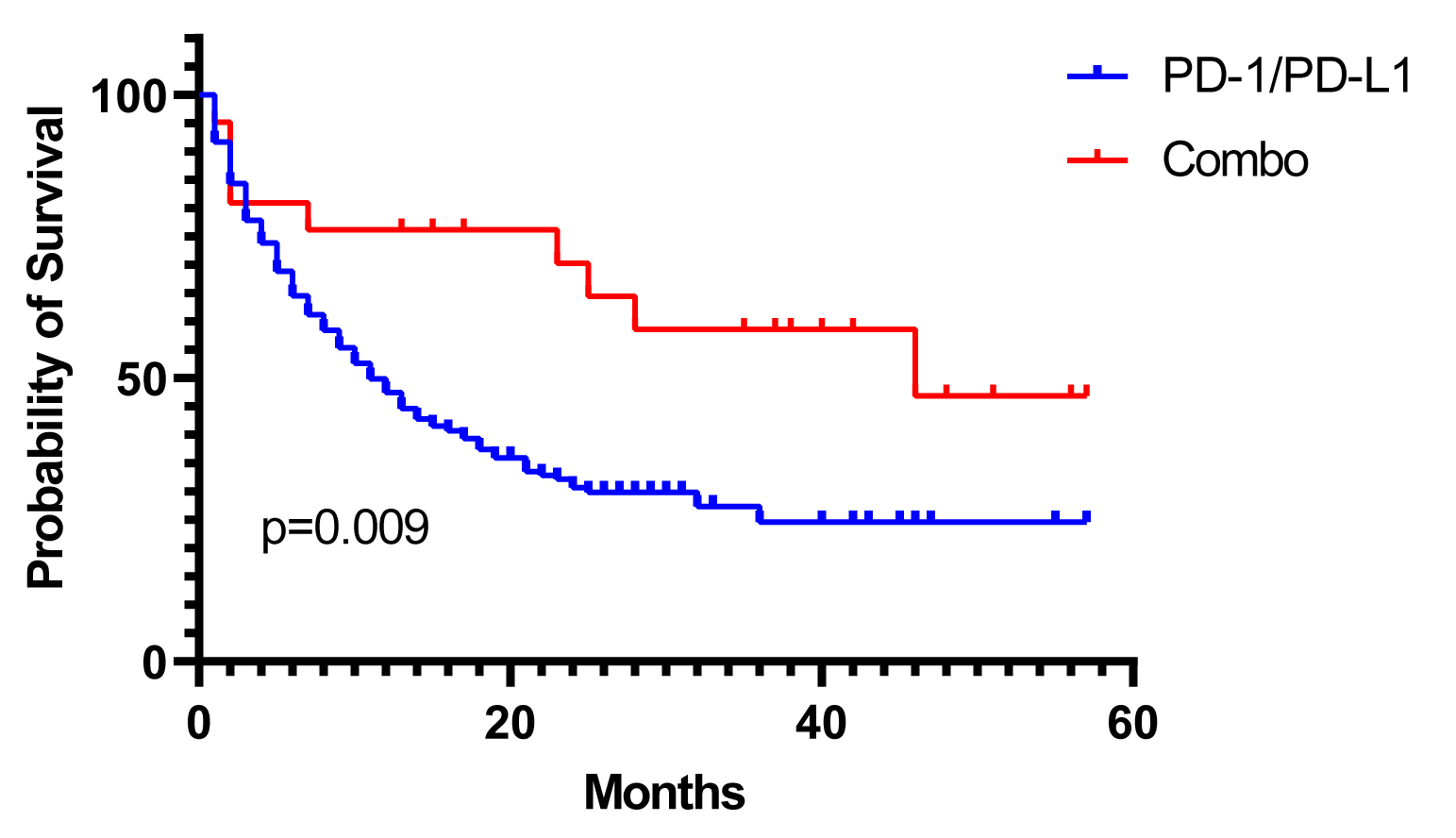

Supplement: Supplementary file 3 — Figure S3 [file CAM4-10-7360-s004.tif]
